# Experimental evaluation does not reveal a direct effect of microRNA from the callipyge locus on DLK1 expression

**DOI:** 10.1186/1471-2164-15-944

**Published:** 2014-10-30

**Authors:** Huijun Cheng, Xuewen Xu, Tracy Hadfield, Noelle Cockett, Carole Charlier, Michel Georges, Haruko Takeda

**Affiliations:** Unit of Animal Genomics, GIGA-R & Faculty of Veterinary Medicine, University of Liège (B34), 1 Avenue de l’Hôpital, 4000 Liège, Belgium; Key Lab of Agricultural Animal Genetics, Breeding and Reproduction of Ministry of Education, College of Animal Science & Technology, Huazhong Agricultural University, Wuhan, 430070 P. R. China; Department of Animal, Dairy and Veterinary Sciences, Utah State University, Logan, UT 84322 USA

**Keywords:** Callipyge, Muscle development, Polar overdominance, DLK1, MicroRNA, Trans-effect, Post-transcriptional gene regulation, Reporter assay

## Abstract

**Background:**

Polar overdominance at the ovine callipyge (*CLPG*) locus involves the post-transcriptional *trans*-inhibition of *DLK1* in skeletal muscle of *CLPG/CLPG* sheep. The abundant maternally expressed microRNAs (miRNAs) mapping to the imprinted *DLK1-GTL2* domain are prime candidate mediators of this *trans*-effect.

**Results:**

We have tested the affinity of 121 miRNAs processed from this locus for *DLK1* by co-transfecting COS1 cells with a vector expressing the full-length ovine DLK1 with corresponding mimic miRNAs. None of the tested miRNAs was able to down regulate DLK1 to the extent observed *in vivo*.

**Conclusions:**

This suggests that other factors, with or without these miRNAs, are involved in mediating the observed *trans*-effect.

**Electronic supplementary material:**

The online version of this article (doi:10.1186/1471-2164-15-944) contains supplementary material, which is available to authorized users.

## Background

The callipyge phenotype is a muscular hypertrophy that is exclusively expressed by heterozygous sheep inheriting the *CLPG* mutation from their sire (genotype *+* ^*Mat*^*/CLPG*^*Pat*^). This non-Mendelian mode of inheritance with parent-of-origin effect is referred to as polar overdominance [1, 2]. The underlying increase in the proportion and size of fast twitch muscle fibers is thought to be caused by ectopic expression of DLK1 and/or PEG11 (also known as RTL1) protein in skeletal muscle of *+* ^*Mat*^*/CLPG*^*Pat*^ animals. This ectopic expression results from the inactivation – by the *CLPG* A to G point mutation – of a muscle-specific silencer element that post-natally downregulates the expression of *DLK1* and *PEG11* in *cis* [3–6]. *CLPG*^*Mat*^*/+*^*Pat*^ animals do not express the phenotype because *DLK1* and *PEG11* genes are imprinted and only expressed from the paternal allele. It is thought that *CLPG/CLPG* animals do not express the phenotype because of the additional ectopic expression of non-coding RNAs (ncRNAs) that are (i) imprinted and expressed from the maternal allele, (ii) controlled in *cis* by the same muscle-specific silencer element, and (iii) post-transcriptionally down-regulating *DLK1* and *PEG11* in *trans* [7]. Maternally expressed ncRNA genes in the *CLPG* locus include four long ncRNAs (lncRNA) (*GTL2, anti-PEG11, RIAN* and *MIRG*) as well as 110 and 47 embedded miRNAs and C/D snoRNAs, respectively [8–10]. For *PEG11* the *trans*-inhibition results from RNA-induced silencing complex (RISC)-mediated cleavage of *PEG11* transcripts by at least three perfectly complementary miRNAs processed from the maternally expressed *anti-PEG11* lncRNA [11]. For *DLK1*, the mechanism of the *trans*-inhibition remains unknown. The present working model for the callipyge phenomenology is summarized in Additional file 1: Figure S1.

When compared to *+* ^*Mat*^*/CLPG*^*Pat*^ animals, DLK1 levels in *CLPG/CLPG* animals are more severely reduced at the protein (~10-fold) than at the mRNA (~3-fold) level [5, 12, 13]. This pattern is compatible with a miRNA-mediated effect that would affect mRNA stability *and* translation. It made the abundant miRNAs from the *DLK1-GTL2* domain prime candidate mediators of the observed *trans*-inhibition. However, bioinformatic analyses did not reveal a striking affinity of any of these miRNAs for *DLK1.* At best, there was some evidence suggesting that they might have an unusual affinity for the open reading frame (ORF) of *DLK1* when assumed to act jointly as a team [10]. As bioinformatic target predictions reputably have limited sensitivity and specificity, we wanted to experimentally evaluate the effect of the miRNAs from the *DLK1-GTL2* domain on ovine DLK1.

## Results

Given the prior evidence that (i) the miRNA from the *DLK1-GTL2* domain might target the ORF of *DLK1* rather than its 3’ untranslated region (UTR) [10], and (ii) that their effect might be more pronounced on protein than mRNA concentrations [5], we decided to develop a reporter assay that would (i) express full-length ovine *DLK1* transcripts including 5’ UTR, ORF and 3’ UTR, and (ii) assay protein rather than mRNA levels. We first performed 5’ and 3’ rapid amplification of cDNA ends (RACE) experiments using RNA from sheep skeletal muscle to accurately map the predominant transcription start and poly-adenylation sites of *DLK1* (see Additional file 1: Figure S2). We then assembled a 1,405-bp fragment corresponding to 209-bp of ovine upstream sequence (encompassing the transcription start site mapped 174-bp upstream of the ATG start codon), 954-bp of ORF corresponding to the ovine *DLK1* C2 isoform appended with a 9-residue human influenza haemagglutinin (HA) carboxyterminal tag, and 242-bp of downstream sequences including an *Xho*I restriction site (6-bp) and the 236-bp ovine *DLK1* 3’ UTR (ending exactly at the position of the ovine poly-adenylation site)(see Additional file 1: Figure S3A; Additional file 1: Text S1). We selected the C2 isoform of *DLK1* as (i) it is at least 100 times more abundant than the A isoform in ovine skeletal muscle between -2 and +8 weeks relative to birth, and (ii) the ratio of the transcript levels of the C2 and A isoforms is not affected by *CLPG* genotype (implying that both isoforms encompass the determinants required for the *trans*-effect)[5, 14]. The HA tag was introduced because available anti-human DLK1 antibodies did not effectively recognize ovine DLK1 (data not shown). The corresponding fragment was cloned in a slightly modified pcDNA3.1(-) vector, 1-bp downstream of the vector-specific transcriptional start site and 30-bp upstream of the vector-specific bovine growth hormone polyadenylation site. The resulting construct (p3.1M-*DLK1*-HA) was completely sequenced to confirm its integrity. It was shown, upon transfection in COS1 cells, (i) to generate a transcript of expected size (~1.4-kb) (Northern blotting), (ii) bounded by the vector-specific transcription start and polyadenylation sites (and hence appended with 1-base of upstream and 30-bases of downstream vector-specific sequences) (5’ and 3’ RACE), and (iii) producing a protein of expected size recognized by anti-HA antibodies (~34 kDa after deglycosylation, Western blotting) (see Additional file 1: Figure S3). Within the examined range, DLK1 amounts estimated by phosphorimaging and densitometry increased linearly with loaded quantities (r^2^ = 0.98) (see Additional file 1: Figure S4).

We designed 121 mimic miRNAs, corresponding to one or two isomirs for 108 of the 110 miRNA species from the *DLK1-GTL2* domain detected in ovine muscle [10] (see Additional file 2: Table S1 and Methods). In addition, we designed one perfectly complementary anti-*DLK1* siRNA as positive control and two miRNAs, corresponding to scrambled miR-127 and miR-377, as negative controls (hereafter referred to as siRNA, NC1, and NC2, respectively).

**Figure 1:**
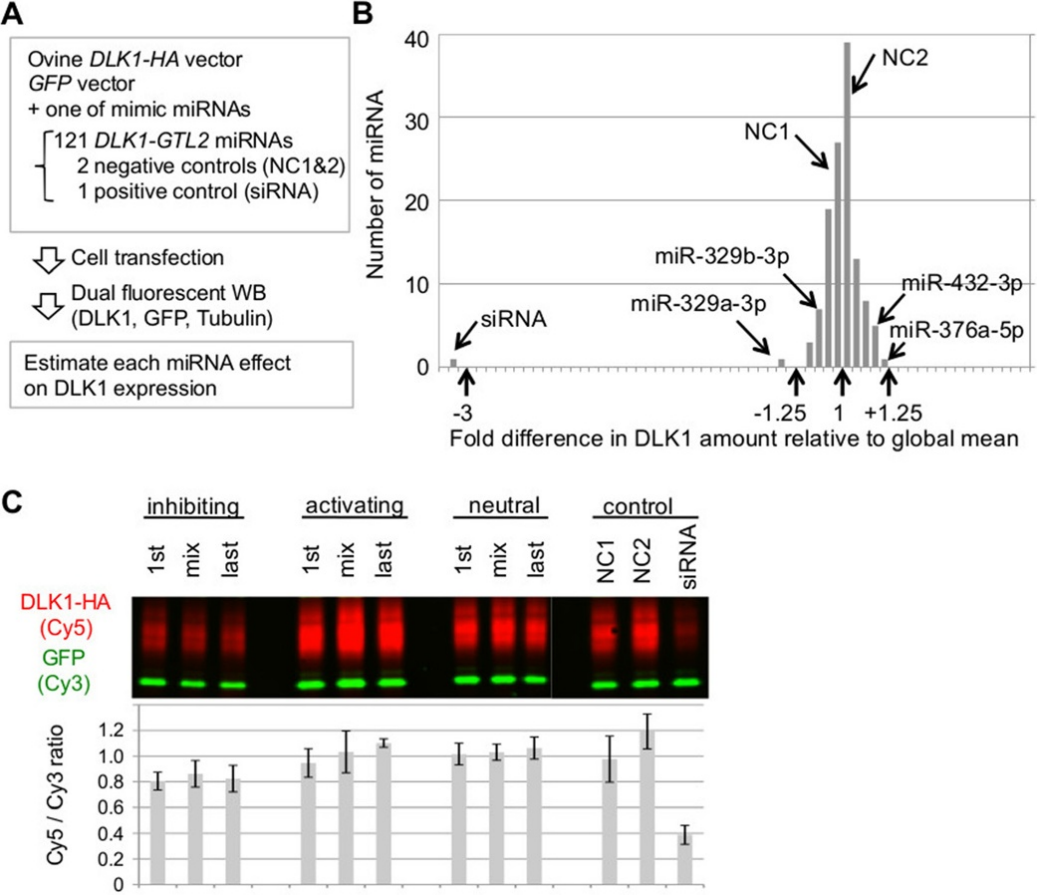
**Experimental evaluation of the effect of microRNA from the callipyge locus on DLK1 expression. (A)** Overview of experimental procedure of ovine DLK1 reporter assay. We co-transfected COS1 cells with a mixture of ovine full-length (5’ UTR, ORF, and 3’ UTR) *DLK1*-expressing vector (p3.1M-*DLK1*-HA), *GFP*-expressing vector (pcDNA3-*GFP*) for normalizing transfection efficiency and gel-loaded protein amount, and one of 121 mimic miRNA candidates from the *DLK1-GTL2* imprinted domain, two negative controls (NC1, NC2), and one siRNA targeting the *DLK1* ORF as positive control. To facilitate protein quantification, an HA epitope was engineered at the carboxyterminal end of DLK1. Amounts of DLK1, GFP, and endogenous Tubulin were estimated by dual fluorescent Western blot (WB). Effect of each miRNA on DLK1 translation was estimated by normalizing DLK1 with either GFP or Tubulin amounts (corrected DLK1 amount). **(B)** Distribution of effects of the 121 mimic miRNAs on DLK1 protein amounts. Effects are expressed as fold difference with respect to the overall (minus siRNA) mean. The effects of two negative (NC1, NC2) and one positive (siRNA) controls are shown, as are the effects of the most inhibiting miR-329a-3p, the closely related miR-329b-3p, and the most activating miR-376a-5p and miR-432-3p. GFP was used for normalization. **(C)** Evaluation of synergistic action of multiple miRNA on DLK1 amount. We selected seven miRNAs consistently showing inhibiting, activating, and neutral effects on DLK1 amount. COS1 cells were co-transfected with the p3.1M-*DLK1*-HA and pcDNA3-*GFP* vectors along with either a mixture of the seven miRNAs (mix), or a single miRNA having shown the lowest (1st) or highest (last) corrected DLK1 amount upon transfection in each group. DLK1-HA and GFP protein levels were estimated by dual fluorescent WB analysis (upper panel). Means of corrected DLK1 amounts with standard deviations for four independent experiments are shown (lower panel).

We transfected COS1 cells with a mixture of DLK1-expressing p3.1M-*DLK1*-HA vector, GFP-expressing pcDNA3-*GFP* vector (to evaluate and correct for variation in transfection efficiency and gel-loaded protein amount), and individual mimic miRNA (Figure 1A). We performed ≥4 independent transfections for each miRNA, and ran ≥2 independent Western blot experiments per transfection, yielding an average of 8.3 usable measures per miRNA. DLK1 amounts, estimated by densitometry, were normalized to GFP amounts as detailed in Methods (see Additional file 1: Figure S5).

As expected (because of its perfect complementarity and predicted cleavage activity), the siRNA positive control exhibited a strong inhibitory effect. It behaved as a complete outlier, reducing the amount of DLK1 ~ 3-fold when compared to the average of the two negative controls, and this effect was highly significant (p = 2 × 10^-16^) (Figure 1B and see Additional file 2: Table S2). Seven of the 121 tested miRNAs significantly (p ≤0.05/121 = 4 × 10^-4^) altered DLK1 levels when compared to the average of the negative controls. One of these caused a reduction in DLK1 levels (miR-329a-3p, 1.25-fold reduction, p = 3.8 × 10^-4^), while the remaining six increased amounts of DLK1 (≤1.28-fold increase, p ≥1 × 10^-8^). Overall, the 121 miRNAs and the two negative controls appeared to belong to one population (Figure 1B). Accordingly, when compared to the average of all miRNAs (except for the siRNA), one miRNA significantly decreased DLK1 levels (miR-329a-3p, 1.31-fold reduction, p = 3.2 × 10^-5^), and two significantly increased DLK1 levels (miR-376a-5p, 1.25-fold increase, p = 5 × 10^-7^; miR-432-3p, 1.18-fold increase, p = 1.8 × 10^-5^). MiR-329b-3p, which differs from miR-329a-3p at only two residues near the 3’ end, reduced DLK1 amounts 1.1-fold when compared to the overall average, but this was not significant when accounting for multiple testing (p = 0.047). MiR-329a-3p and miR-329b-3p have two 6-mer matches in the ORF and one in the 3’ UTR. However, neither of these is evolutionary conserved (see Additional file 1: Figure S6). miR-329a-3p and miR-329b-3p accounted respectively for 0.0014% and 0.19% of miRNA molecules derived from the *DLK1-GTL2* domain and 0.00043% and 0.056% of total miRNA molecules in skeletal muscle of *CLPG/CLPG* animals [10]. It is also noteworthy that we did not observe a convincing correlation between effect on DLK1 levels and bioinformatically predicted affinity for *DLK1*, whether using seed-based scores [15] or Miranda [16], and whether considering the 3’ UTR, the ORF, the 5’ UTR or the full-length *DLK1* transcript (see Additional file 1: Figure S7; Additional file 2: Table S2).

Taken together, these data suggest that none of the tested miRNAs effectively regulates DLK1. Although the effect of miR-329a-3p is significant, we consider the evidence insufficient to claim that it is biologically relevant. Indeed, (i) the effects of the 121 tested miRNAs are approximately normally distributed, encompass the effects of the two negative controls, and are not correlated with predicted affinity for *DLK1*, (ii) there are more miRNAs that have a significant positive effect than miRNAs that have a negative effect on DLK1 expression (which is unlikely to reflect a genuine biological activity given the known *modus operandi* of miRNAs), (iii) the effect of miR-329a-3p becomes insignificant when considered jointly with the closely related miR-329b-3p, (iv) the expression level of miR-329a-3p in ovine skeletal muscle is extremely low. What is certain is that none of the tested miRNAs is capable – under the utilized conditions - to single-handedly downregulate DLK1 to the extent that is observed *in vivo* in skeletal muscle of *CLPG/CLPG* sheep.

It remains possible that the observed *trans*-inhibition of *DLK1* results from the synergistic action of multiple miRNAs. Although we could obviously not test all combinations of miRNAs, we transfected COS1 cells with pools of seven miRNAs chosen (i) amongst the most inhibiting, and (ii) most activating miRNAs. The pools of extremes inhibited, respectively enhanced, DLK1 expression to a degree similar to their constituent miRNAs, but did not reveal convincing evidence for a synergistic effect (Figure 1C and see Additional file 1: Figure S8).

## Discussion

The aim of this study was to experimentally verify whether any of the miRNAs processed from the ovine *DLK1-GTL2* domain might post-transcriptionally down-regulate DLK1 to a degree that is commensurate with what is observed in skeletal muscle of *CLPG/CLPG* sheep. Although one miRNA (miR-329a-3p) yielded an effect deemed significant even when accounting for multiple testing, it was not considered to be relevant with regards to the *trans*-inhibition of DLK1 in *CLPG/CLPG* animals for the reasons given above. Of note, when performing ANOVA analysis the “miRNA effect” (not including the three controls) was highly significant (p <2 × 10^-16^). Thus, the effects of at least some miRNAs are repeatable despite being modest. This could reflect subtle effects on DLK1, on GFP, or on the cellular machinery.

Negative results are in essence uncomfortable to interpret. They may be false negatives because the established reporter assay misses an essential component that is required for the responsible miRNAs to exert their effect. MiRNA-mediated regulation of DLK1 may require auxiliary *trans*-acting factors not expressed in COS1 cells. The expressed transcripts corresponded for >93% to the ovine *DLK1* C2 mRNA, yet included 36-bases of extra upstream, 33-bases of extra internal (HA tag), and 30-bases of extra downstream sequence, and these may have affected miRNA-target interactions. To discriminate between a miRNA-dependent versus independent mechanism, we have conducted immuno-precipitation experiments using antibodies directed against the Argonaut components of the RISC [17], hoping to show an increase in *DLK1* co-immunoprecipitation in skeletal muscle of *CLPG/CLPG* when compared to *+* ^*Mat*^*/CLPG*^*Pat*^ animals, but have so far failed to obtain convincing results (data not shown).

Alternatively, our results may be true negatives and indicate that the *trans*-inhibition of *DLK1* observed *in vivo* is not a post-transcriptional regulation mediated directly by miRNAs processed from the *DLK1-GTL2* domain. It is worthwhile noting in this regard that, while the larger effect on DLK1 protein than on *DLK1* transcripts is reminiscent of early reports of the *modus operandi* of the archetypical animal miRNA *lin-4* on its *lin-28* and *lin-14* targets in *C. elegans*, *lin-4*’s mode of action has since been revisited and shown to be more in line with the canonical one in which mRNA destabilization accounts for the major component of repression [18, 19]. Moreover, the ~3-fold and ~10-fold down-regulation of DLK1 observed respectively at the mRNA and protein levels *in vivo* would be rather extreme for miRNA-dependent effects [20, 21]. Alternative hypotheses include a direct post-transcriptional effect of one or several of the maternally expressed lncRNAs, or an indirect post-transcriptional effect of anyone of the ncRNAs from the domain. As for most lncRNAs, little is known about the function of the maternally expressed *GTL2*, *RIAN* and *MIRG* genes. It has been suggested that *GTL2* recruits Polycomb Repressive Complex 2 (PRC2) to down-regulate the reciprocally imprinted *DLK1* gene in *cis* [22]. Preliminary results of the co-transfection of COS1 cells with p3.1M-*DLK1*-HA with a vector expressing the predominant isoform of ovine *GTL2* (corresponding to human *GTL2* variant 1, NR_002766.2) does not support a direct inhibitory *trans*-effect of *GTL2* on DLK1 expression, on the contrary (see Additional file 1: Figure S9). The hypothesis of an indirect effect posits that the maternally expressed ncRNAs affect targets that do not originate from the *DLK1-GTL2* locus, and that these in turn affect DLK1 expression. Such indirect effects may manifest themselves by changes in transcript levels that could be studied by comparing the transcriptome (including small RNAs) of *CLPG*^*Mat*^*/+*^*Pat*^ with that of +/+ animals, and efforts towards that goal are in progress. Along similar lines, Bidwell *et al*. [23] recently showed that muscle of *CLPG/CLPG* animals exhibited lower expression of a group of key regulatory genes for muscle development (e.g. *ZFP106, RPS6KA3, MEF2A*), that might render them less responsive to the hypertrophic stimuli of the paternal allele-specific genes.

The apparently stronger effect on *DLK1* protein (~10-fold) than on *DLK1* transcript (~3-fold) level suggests a post-transcriptional mechanism. This hypothesis received considerable support from the demonstration of the RISC-mediated cleavage of *PEG11* by miRNAs processed from anti-*PEG11* [11]. However, we cannot totally exclude a transcriptional mechanism. It may be worthwhile, in this regard, to perform ribosome profiling [24] experiments to verify whether the predicted translational inhibition of *DLK1* transcripts in *CLPG/CLPG* animals when compared to *+* ^*Mat*^*/CLPG*^*Pat*^ animals is indeed observed. Lack of such effect would force us to re-evaluate scenarios involving transcriptional regulation.

More work will be needed to gain a full understanding of the molecular mechanisms underlying the *trans*-inhibition of *DLK1*, which is key element of polar overdominance at the *CLPG* locus.

## Conclusions

Contrary to that of *PEG11*, the trans-inhibition of *DLK1* that is observed in *CLPG/CLPG* sheep and lies at the heart of polar overdominance, may not depend on post-transcriptional regulation by miRNAs from the *DLK1-GTL2* domain.

## Methods

### Ethical statement

All animal procedures were carried out in strict accordance with the recommendations in the Guide for the Care and Use of Laboratory Animals of Utah State University and approved by the Animal Ethics Committees of Utah State University (IACUC #386).

### RACE

The 5’ and 3’ ends of *DLK1* transcripts were determined using the GeneRacer Kit (Invitrogen, Carlsbad, CA) according to the manufacturer’s instructions. Briefly, total RNA was isolated either from skeletal muscle (*longissimus dorsi*) of 2-week-old sheep of the four possible *CLPG* genotypes (*+/+, +*^*Mat*^*/CLPG*^*Pat*^*, CLPG*^*Mat*^*/+*^*Pat*^*, CLPG/CLPG*), or from COS1 cells 24 hrs after transfection with the ovine *DLK1* expressing p3.1M-*DLK1*-HA vector. For 5’ RACE, 3 μg of total RNA was used for ligation of the GeneRacer RNA Oligo to the 5’ end of RNA with a 5’ cap structure. RNA was then reverse transcribed using SuperScript III reverse transcriptase using random hexamers. For 3’ RACE, cDNA was synthesized using the GeneRacer Oligo dT primer. These cDNAs were then amplified with the GeneRacer 5’ and *DLK1*-specific 5’ GSP-A primers for 5’ RACE, and the GeneRacer 3’ and *DLK1*-specific 3’ GSP-S primers for 3’ RACE, respectively. Nested PCR amplifications were performed using diluted PCR products with the GeneRacer 5’ nested and *DLK1*-specific 5’ NGSP-A primers for 5’ RACE, and the GeneRacer 3’ nested and *DLK1*-specific 3’ NGSP-S primers for 3’ RACE, respectively. For both 5’ RACE PCRs, we used the Expand Long Template PCR System (Roche Diagnostics, Basel, Switzerland) with PCR conditions: 94°C for 2 min; 10 cycles of 94°C for 10 sec, 62°C for 30 sec and 68°C for 1 min; 22 cycles of 94°C for 15 sec, 62°C for 30 sec and 68°C for 1 min; and 68°C for 7 min. We used the Phusion DNA polymerase (Finnzymes*,* Espoo, Finland) for the 3’ RACE PCRs using touchdown PCR condition: 98°C for 30 sec; 8 cycles of 98°C for 15 sec, 66°C for 20 sec and 72°C for 40 sec; 20 cycles of 98°C for 15 sec, 62°C for 20 sec and 72°C for 40 sec; and 72°C for 3 min. The nested PCR products were analyzed by agarose gel electrophoresis, purified from the gel, and sequenced either directly or after subcloning in the pCR2.1 TA cloning vector (Invitrogen) to determine the 5’ and 3’ ends of the *DLK1* transcripts. All primer sequences are listed in Additional file 2: Table S3.

### Generation of the p3.1M-*DLK1-HA*DLK1 expression vector

The ovine *DLK1* ORF (C2 isoform) was PCR-amplified using ovine skeletal muscle cDNA with primers p3.1-CS and p3.1-CA containing *Xba*I and *Xho*I sites at their 5’ ends, respectively. The reverse primer (p3.1-CA) included a HA tag sequence (AGCGTAGTCTGGGACGTCGTATGGGTA) to add the HA epitope at the carboxyterminus of ovine DLK1 protein. The *Xba*I/*Xho*I double-digested PCR fragment was cloned into the *Xba*I/*Xho*I sites of the pcDNA3.1(-) mammalian expression vector (Invitrogen) to create an intermediate vector p3.1-*DLK1*-CDS-HA. The 5’ UTR and a part of the coding sequence of ovine *DLK*1 was amplified from genomic DNA using the Expand Long Template PCR system with primers p3.1-5S containing an *Xba*I site at the 5’ end and p3.1-5A encompassing an endogenous *Nco*I site in the middle of the primer. The ovine *DLK1* 3’ UTR was PCR-amplified using genomic DNA with primers, p3.1-3S and p3.1-3A containing *Xho*I and *EcoR*I sites at their 5’ ends, respectively. The two resultant PCR fragments were double-digested with *Xba*I/*Nco*I and *Xho*I/*EcoR*I, respectively, and inserted sequentially into the p3.1-*DLK1*-CDS-HA vector at the corresponding sites to generate the p3.1-*DLK*1-FL-HA vector. To minimize vector sequence in the transcripts (more specifically the multiple cloning region between the transcription start and end sites of the vector), we modified the original pcDNA3.1(-) vector (referred to as p3.1M). We introduced an *Xba*I recognition site adjacent to the putative transcriptional start site and an *EcoR*I site upstream of the bovine growth hormone polyadenylation signal of the vector using the Quickchange lightening site-directed mutagenesis kit (Stratagene, La Jolla, CA) with oligonucleotides pc3.1-*Xba*I-intro-U/D and pc3.1-*EcoR*I-intro-U/D, respectively. The insert of the p3.1-*DLK*1-FL-HA (containing the HA tagged full-length of ovine *DLK1*) was excised by an *XbaI/EcoRI* double-digestion and transferred to the modified p3.1M vector at the corresponding sites to generate the final p3.1M-*DLK*1-HA vector. Orientation and integrity of the 1,405-bp insert was confirmed by sequencing using the BigDye Terminator v3.1 Cycle Sequencing kits and the 3730 DNA Analyzer (Applied Biosystems, Foster City, CA) in three sequencing reactions using primers, Seq-S, Seq-A, and 3’ NGSP-S. Primer information is available in Additional file 2: Table S3.

### Mimic miRNAs – choice of the system

To co-express miRNAs from the *DLK1-GTL2* domain with the vector expressing *DLK1*, we initially elected to clone the corresponding miRNA precursors in expression vectors and co-transfect them. The hope was that one vector would allow for the expression of multiple miRNA species and isomirs (one miRNA precursor can give rise to two miRNA “species” (from the 5p and 3p arms, respectively), that can each come in multiple forms or “isomirs”), and that this would optimally mimic the condition prevailing *in vivo* at minimum cost. We realized, however, that the expression levels of the different miRNA species varied enormously, without necessarily mimicking the *in vivo* ratios [10], at concentrations that would often be <1% of endogenous *let7d* levels (data not shown). As this would make comparisons between miRNAs nearly impossible, we opted for the use of synthetic “mimic” miRNAs.

### Mimic miRNAs – molecular design

We evaluated the efficacy of different designs of mimic miRNAs using the luciferase assay described in Takeda *et al*. [17] (see Additional file 1: Figure S10). It is based on the pRL-4xTex and pRL-4xRom vectors, that each contains four tandem repeats respectively with and without miR-1 target sites. We compared four types of mimic miRNAs: (i) an unmodified RNA duplex corresponding to the endogenous mature and star ovine miR-1 miRNAs (hence with bulge, wobble base pairs and mismatch), (ii) an unmodified RNA duplex corresponding to the mature ovine miR-1 and its perfect reverse complement, with 2-nt 3’ overhangs at both ends (UU for the passenger strand), (iii) the same as in (ii) but with modified 5’ ends aimed at promoting the incorporation of the guide strand into the RISC complex: 5’ P (phosphate) for the mature miRNA strand and/or 5’ NH_2_ for the passenger strand, and (iv) the proprietary miR-1 miRNA Precursor purchased from Ambion. While type (i) (i.e. a copy of the endogenous double-stranded miRNA) did not have any effect, types (ii), (iii) and (iv) worked equally well. We opted for type (ii) with unmodified mature strand and 5’ NH_2_ modified passenger strand. The 3’ overhangs for all passenger strands corresponded to UU dinucleotides. Corresponding oligonucleotides were purchased from GenePharma (Shanghai, China).

### Mimic miRNAs – sequence determination

To select representative mimic miRNA sequences for all miRNAs from the *DLK1-GTL2* domain, we simultaneously utilized (i) previously generated RNA-Seq information that was used to establish a catalogue of miRNAs expressed in ovine skeletal muscle with special emphasis on the *DLK1-GTL2* domain [10], (ii) miRNA characteristics inferred from meta-analyses of mouse and Drosophila melanogaster small RNAs [25, 26], and (iii) information of human, mouse, and bovine orthologs from miRBase [27]. In most cases, the selected sequences corresponded to the most abundant isomirs in the ovine RNA-Seq data, matching the miRBase registered sequences, and formed a canonical mature and star miRNA duplex with 3’ overhangs. To those, we added (i) “star” sequences that were not registered in the miRBase but were observed in our RNA-Seq data, and (ii) alternative isomir with 5’ heterogeneity that represented more than one third of the reads of a corresponding miRBase-registered miRNA or with more than 200 reads in any of the RNA-Seq libraries. A-to-I editing [10] or untemplated nucleotide addition (data not shown) were observed in some of miRNAs from the *DLK1-GTL2* domain. This was accounted for by synthesizing isomirs with mixed bases at the corresponding sites. The complete list of 121 tested mimic miRNAs is provided in Additional file 2: Table S1. MiRNAs derived from the 5p and 3p arms of a miRNA hairpin precursor are suffixed with -5p and -3p, respectively, irrespective of their relative expression levels.

To design negative control miRNA, we first shuffled sequences of five miRNAs (oar-miR-433-3p, 127-3p, 3957-3p, 377-3p, and 376c-3p) ten times each. We then counted the number of 8-mer, 7mer-A1, 7mer-m8, and 6-mer seed matches [15] of these shuffled sequences to the full-length ovine *DLK1* C2 isoform. In addition, to minimize off-target effects on unintended endogenous RNAs, we predicted the number of genome wide conserved miRNA target sites across most mammals using the TargetScan Custom software [28, 29]. Two of the shuffled sequences that showed neither 6-, 7- nor 8-mer seed matches to the ovine *DLK1* and the lowest number of predicted genome-wide conserved target sites (seven and one conserved targets, respectively) were selected as negative controls (designated as Scr-oar-miR-127-3p (NC1) and Scr-oar-miR-377-3p (NC2)). In addition, we purchased one siRNA targeting the ovine *DLK1* ORF by using the BLOCK-iT RNAi Designer (Invitrogen), to be used as positive control.

### Cell culture and transient transfection

COS1 cells were cultured in Dulbecco’s modified Eagle’s medium supplemented with 10% fetal bovine serum and 0.1 mM non-essential amino acids, 100 units/ml penicillin and 100 mg/ml streptomycin at 37°C (GIBCO, Renfrewshire, UK). One day before transfection, 0.5 × 10^5^ cells were seeded in a 12-well plate in 800 μl of growth medium without antibiotics. We then co-transfected the cells with 200 μl of Opti-MEM medium (GIBCO) containing 200 ng of the p3.1M-*DLK1*-HA vector, 400 ng of pcDNA3-*GFP* vector (Plasmid ID: 13031 obtained from Addgene, Cambridge, MA) along with 40 pmols of synthetic mimic miRNA (GenePharma)(final concentration 40 nM) or 5 pmols of siRNA (Invitrogen) using 4 μl of Lipofectamin 2000 (Invitrogen) according to the manufacturer’s instructions.

### Dual fluorescence Western blotting

Total protein was extracted from COS1 cells 24 hrs after transfection using a modified RIPA buffer containing 50 mM Tris-HCl (pH 7.6), 150 mM NaCl, 1% Igepal CA-630, 0.25% sodium deoxycholate, 1 mM EDTA and the complete protease inhibitor cocktail (Roche). The lysate was centrifuged at 12,000 × g for 15 min at 4°C and protein concentration of the supernatant was determined using the micro BCA Protein Assay Kit (Pierce*,* Rockford, IL) according to the manufacturer's instructions. Eight μg of protein were boiled for 5 min in Laemmli buffer (62.5 mM Tris-HCl (pH 7.6), 2% sodium dodecyl sulfate (SDS), 10% glycerol, 0.004% bromophenol blue, 5% β-mercaptoethanol) and loaded on a NuPAGE Novex 4-12% Bis-Tris Midi gel (Invitrogen). Electrophoresis was performed with the XCell4 SureLock Midi-Cell electrophoresis chamber (Invitrogen) at 200 V for 30 min at room temperature in the NuPAGE MOPS SDS Running buffer (Invitrogen). Protein was then transferred to a Hybond-LFP Polyvinylidene difluoride (PVDF) membrane (GE Healthcare, Buckinghamshire, UK) at 200 mA for 90 min at 4°C using the NuPAGE transfer buffer (Invitrogen) in a Criterion Blotter (BioRad, Hercules, CA). The membrane was blocked with 10% non-fat dry milk in PBST buffer (1 mM KH_2_PO_4_, 10 mM Na_2_HPO_4_, 137 mM NaCl, 2.7 mM KCl, 0.1% Tween 20) either at room temperature for 1 hr or at 4°C overnight. The membrane was incubated sequentially with a series of antibodies diluted in the blocking buffer at room temperature for 1 hr. We used a rabbit polyclonal anti-HA tag antibody (1:4000 dilution) (ab9110, Abcam, Cambridge, UK), an ECL Plex anti-rabbit IgG antibody conjugated with Cy5 dye (1:2500) (PA45011, GE Healthcare), a mouse monoclonal anti-GFP antibody (1:1000)(clone JL-8, Clontech Laboratories, Mountain View, CA), a mouse monoclonal anti-α-Tubulin antibody (1:8000) (T6074, Sigma, St. Louis, MO), and an ECL Plex anti-mouse IgG antibody conjugated with Cy3 dye (1:4000) (PA43010, GE Healthcare). Membranes were washed three times for 10 min in PBST buffer with gentle shaking between incubations. The membrane was then dried at 37°C for 1 hr, followed by scanning with the Typhoon 9400 scanner (GE healthcare). Band intensities corresponding to HA-tagged DLK1, GFP, and Tubulin were quantified using the ImageQuant TL software (GE healthcare). For normalization, the band intensities were first divided by the corresponding membrane-specific averages (not including siRNA), yielding “relative” DLK1, GFP, Tubulin amounts. The relative DLK1 amounts were then divided by either the corresponding relative GFP or Tubulin amounts, yielding “corrected” DLK1 amounts. We performed more than four independent transfections of cells for each mimic miRNA, and ran more than two independent Western blot experiments per transfection. MiRNA effect on the DLK1 expression was estimated by comparing the mean of the corrected DLK1 amounts for a given miRNA either to the global mean (not including siRNA data) or to the average of NC1 and NC2 data.

### Deglycosylation

As DLK1 has been reported to be *N*- and *O*-glycosylated [30], we carried out Western blot analysis using proteins treated with the Protein Deglycosylation mix (New England Biolabs, Ipswich, MA) according to the manufacturer’s instructions. Briefly, COS1 cells transfected with the p3.1M-*DLK1*-HA vector were harvested in the Phosphate Buffered Saline (GIBCO) supplied with 1% Igepal CA-630 and the complete protease inhibitor cocktail. Twenty μg of protein were denatured at 99°C for 10 min and treated at 37°C for 4 hrs with a mixture of deglycosylation enzymes that were expected to remove all *N*-linked and simple *O*-linked glycans, as well as some complex *O*-linked glycans. Western blot was performed as described above.

### Northern blot analysis

Total RNA was isolated from COS1 cells 24 hrs after transfection with the p3.1M-*DLK1*-HA vector using the Trizol reagent (Invitrogen) according to the manufacturer’s protocol. Ten μg of total RNA was denatured at 50°C for 10 min in 20 μl of 1 × MOPS buffer (20 mM 3-(N-morpholino) propanesulfonic acid, 5 mM sodium acetate, 1 mM EDTA, 1 mM EGTA, pH 7.0) supplied with 2.4 M formaldehyde and 50% formamide. The RNA was mixed with loading buffer (final 5% glycerol, 0.025% bromophenol blue, 0.025% xylene cyanol) and subjected to electrophoresis on a 1% agarose gel containing 1.75 M formaldehyde in 1 × MOPS buffer at 5 V/cm for 3.5 hrs. After electrophoresis, the RNA was transferred onto a Hybond-N^+^ nylon membrane (GE Healthcare) with 10 × SSC buffer (150 mM sodium citrate, 1.5 M NaCl, pH 7.0) and UV cross-linked essentially as described in Sambrook *et al*. [31]. A DNA fragment containing the ovine *DLK1* ORF region (857-bp) was gel-purified after digesting the plasmid p3.1M-*DLK1*-HA with *Nco*I and *Bgl*II and labeled with [α-^32^P] dCTP (GE Healthcare) using the Prime-It II random primer labeling kit (Stratagene). The membrane was pre-hybridized with the ULTRAhyb buffer (Ambion) at 42°C for 2 hrs and then hybridized with 1 × 10^6^ cpm/ml of the ^32^P-labeled probe at 45°C overnight. Membranes were washed twice at 55°C for 20 min in 2 × SSC and 0.1% SDS, then twice at 55°C for 30 min in 0.1 × SSC and 0.1% SDS. Membranes were exposed to the Hyperfilm ECL film (GE Healthcare) for 3 hrs.

### Generation of ovine *GTL2*expression vector

Ovine *GTL2* sequence (see Additional file 1: Text S2) was PCR-amplified from cDNA synthesized with random hexamers from *longissimus dorsi* skeletal muscle RNA of an 8-week-old *CLPG/CLPG* animal using the Phusion DNA polymerase with primers, ovGTL2-FL-S1 and ovGTL2-FL-A1 (see Additional file 2: Table S3). The resultant PCR product was sub-cloned into the pCRII-blunt-TOPO vector (Invitrogen) and subjected to DNA sequencing for confirmation. The insert was excised with *EcoR*I and ligated into the corresponding site of the pcDNA3.1(-) vector to produce the p3.1-*GTL2* and p3.1-*antiGTL2* vectors that express ovine *GTL2* in sense and antisense orientations, respectively. Integrity of the insert was confirmed by sequencing.

### In silico affinity score of miRNA to *DLK1*

Affinity scores of each miRNA for ovine *DLK1* transcript expressed from the p3.1M-*DLK1*-HA were computed using Miranda [16] and a seed-based score [15]. Miranda scores were obtained by running the Miranda v3.3a software using the miRNA sequences (see Additional file 2: Table S1) and the ovine *DLK1* 5’ UTR, ORF, 3’ UTR, and full-length sequences (see Additional file 1: Text S1). For the Grimson seed match score, we simply counted the number of sites complementary to miRNA seed sequences (8mer, 7mer-A1, 7mer-m8, 6mer, and 6mer-offset seed matches [15]) on the *DLK1* sequences. Pearson correlation coefficients between the prediction scores and the corrected DLK1 amounts (DLK1/GFP) (without controls) and its significant levels were calculated using standard procedures.

## Electronic supplementary material

Additional file 1: Figure S1: Working model for polar overdominance at the ovine callipyge locus [11]. **Figure S2**: Characterization of the 5’ and 3’ ends of ovine *DLK1* transcripts in ovine skeletal muscle. **Figure S3**: Construction and characterization of the p3.1M-*DLK1*-HA vector expressing full length ovine *DLK1* [30]. **Figure S4**: Quantitativeness of Western blot analysis of ovine DLK1 protein. **Figure S5**: Dual fluorescent Western blot to measure DLK1 amount. **Figure S6**: Conservation around oar-miR-329a-3p 6-mer seed matches on *DLK1* [15, 32]. **Figure S7**: Weak correlation between miRNA-target affinity score and DLK1 [15, 16]. **Figure S8**: MiRNAs used for a multiple-miRNA transfection test. **Figure S9**: Effect of *GTL2* lncRNA expression on DLK1 amount. **Figure S10**: Functionality of synthetic mimic miRNAs [17]. **Text S1**: RNA sequence transcribed from the p3.1M-*DLK1*-HA expression vector. **Text S2**: Ovine *GTL2* sequence in the p3.1-*GTL2* expression vector. (PDF 2 MB)

Additional file 2: Table S1: Mimic miRNA sequence information [10, 33]. **Table S2**: Effect of each mimic miRNA on DLK1 expression and its correlation with predicted affinity scores [15, 16]. **Table S3**: Oligonucleotides used in this study. (XLSX 86 KB)

## Abbreviations

CLPG: Callipyge
*DLK1*: Delta-like 1 homolog (Drosophila)
*PEG11*: Paternally expressed gene 11
*RTL1*: Retrotransposon-like 1
*GTL2*: Gene trap locus 2
*RIAN*: RNA imprinted and accumulated in nucleus
*MIRG*: MicroRNA containing gene
miRNA: microRNA
ncRNA: non-coding RNA
lncRNA: long non-coding RNA
snoRNA: small nucleolar RNA
dsRNA: double stranded RNA
ORF: Open reading frame
UTR: Untranslated region
PRC2: Polycomb repressive complex 2
RACE: Rapid amplification of cDNA ends
HA: Haemagglutinin
RISC: RNA-induced silencing complex
moR: miRNA-offset RNA.
