# Supplementary material for: Experimental evaluation does not reveal a direct effect of microRNA from the callipyge locus on DLK1 expression
Source: BMC Genomics. 2014 Oct 30;15(1):944. doi: 10.1186/1471-2164-15-944 (PMC4226911; doi:10.1186/1471-2164-15-944)
Supplement: Supplementary file 1 — Additional file 1: Figure S1: Working model for polar overdominance at the ovine callipyge locus [11]. Figure S2: Characterization of the 5’ and 3’ ends of ovine DLK1 transcripts in ovine skeletal muscle. Figure S3: Construction and characterization of the p3.1M-DLK1-HA vector expressing full length ovine DLK1 [30]. Figure S4: Quantitativeness of Western blot analysis of ovine DLK1 protein. Figure S5: Dual fluorescent Western blot to measure DLK1 amount. Figure S6: Conservation around oar-miR-329a-3p 6-mer seed matches on DLK1 [15, 32]. Figure S7: Weak correlation between miRNA-target affinity score and DLK1 [15, 16]. Figure S8: MiRNAs used for a multiple-miRNA transfection test. Figure S9: Effect of GTL2 lncRNA expression on DLK1 amount. Figure S10: Functionality of synthetic mimic miRNAs [17]. Text S1: RNA sequence transcribed from the p3.1M-DLK1-HA expression vector. Text S2: Ovine GTL2 sequence in the p3.1-GTL2 expression vector. (PDF 2 MB) [file 12864_2014_6639_MOESM1_ESM.pdf]

Additional FILE 1 as PDF

Experimental evaluation does not reveal a direct effect of microRNA from the callipyge locus on DLK1 expression.

Huijun Cheng[1, 2], Xuewen Xu[1, 2], Tracy Hadfield[3], Noelle Cockett[3], Carole Charlier[1], Michel Georges[1], Haruko Takeda[1].

[1]Unit of Animal Genomics, GIGA-R & Faculty of Veterinary Medicine, University of Liège (B34), 1 Avenue de l'Hôpital, 4000-Liège, Belgium. [2]Key Lab of Agricultural Animal Genetics, Breeding and Reproduction of Ministry of Education, College of Animal Science & Technology, Huazhong Agricultural University, Wuhan, 430070, P. R. China, [3]Department of Animal, Dairy and Veterinary Sciences, Utah State University, Logan, UT 84322, USA.

Additional file 1: Supplemental Figure 1: Working model for polar overdominance at the ovine callipyge locus.

Additional file 1: Supplemental Figure 2: Characterization of the 5' and 3' ends of ovine *DLK1* transcripts in ovine skeletal muscle.

Additional file 1: Supplemental Figure 3: Construction and characterization of the p3.1M-*DLK1*-HA vector expressing full length ovine *DLK1*.

Additional file 1: Supplemental Figure 4: Quantitativeness of Western blot analysis of ovine DLK1 protein.

Additional file 1: Supplemental Figure 5: Dual fluorescent Western blot to measure DLK1 amount.

Additional file 1: Supplemental Figure 6: Conservation around oar-miR-329a-3p 6-mer seed matches on *DLK1*.

Additional file 1: Supplemental Figure 7: Weak correlation between miRNA-target affinity score and DLK1.

Additional file 1: Supplemental Figure 8: MiRNAs used for a multiple-miRNA transfection test.

Additional file 1: Supplemental Figure 9: Effect of *GTL2* lncRNA expression on DLK1 amount.

Additional file 1: Supplemental Figure 10: Functionality of synthetic mimic miRNAs.

Additional file 1: Supplemental Text 1: RNA sequence transcribed from the p3.1M-*DLK1*-HA expression vector.

Additional file 1: Supplemental Text 2: Ovine *GTL2* sequence in the p3.1-*GTL2* expression vector.

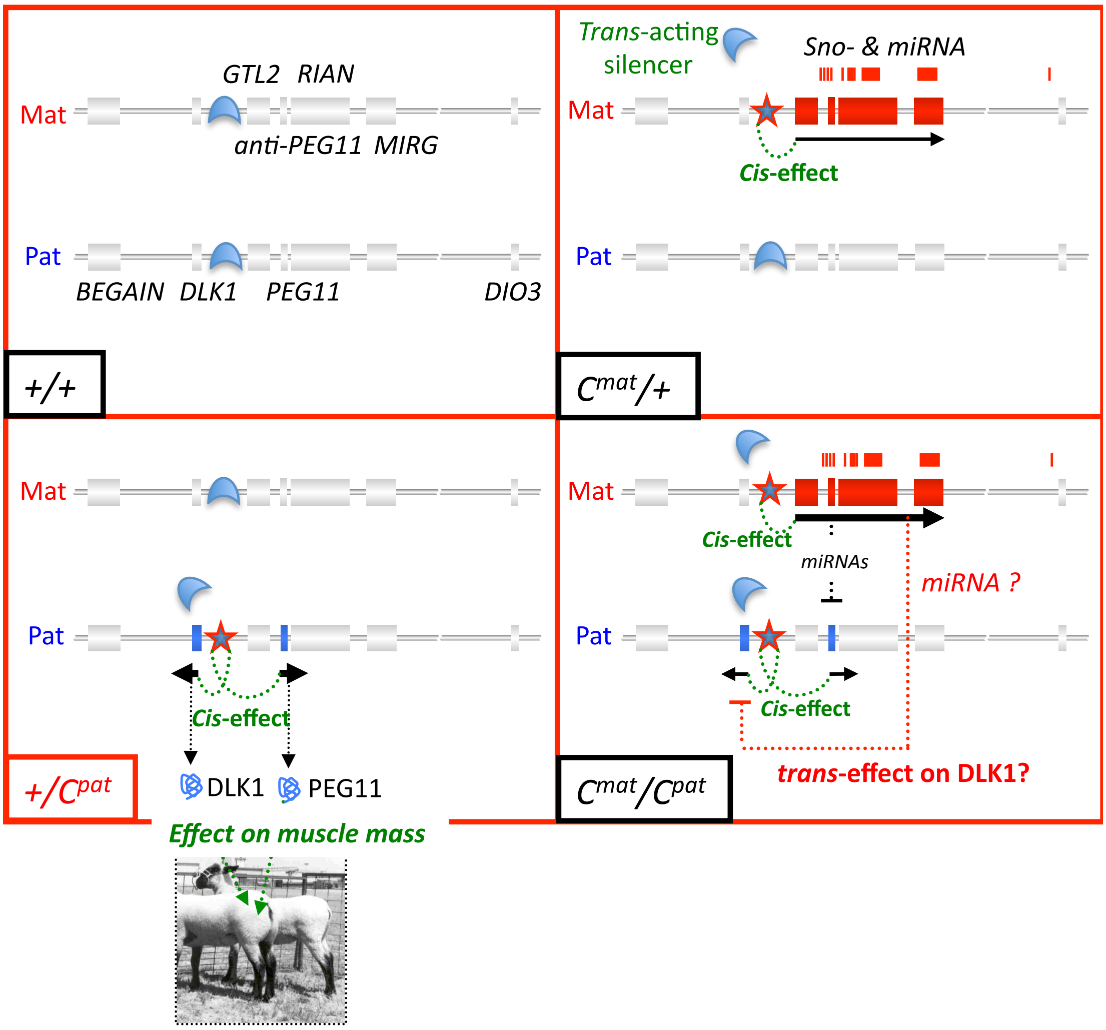

Supplemental Figure 1: Working model for polar overdominance at the ovine callipyge locus. The callipyge phenotype is an inherited muscular hypertrophy of sheep characterized by polar overdominance: only heterozygous individuals inheriting the *CLPG* mutation from their father ($+^{mat}/CLPG^{pat}$) are callipyge. The boxes illustrate the expression profile of imprinted genes in the *DLK1-GTL2* domain in skeletal muscle according to the four possible *CLPG* genotypes ($+/+$, $CLPG^{mat}/+^{pat}$, $+^{mat}/CLPG^{pat}$, $CLPG/CLPG$). We propose (i) that the *CLPG* mutation (shown by a star) inactivates a muscle-specific silencer (binding a trans-acting factor represented by the crescent) that postnatally downregulates the expression of imprinted genes in the *DLK1-GTL2* domain without affecting parental imprinting, (ii) that the callipyge phenotype involves the ectopic expression of the paternally expressed protein encoding *DLK1* and *PEG11* genes and its protein products in skeletal muscle after birth in $+^{mat}/CLPG^{pat}$ animals, and (iii) that polar overdominance results from the trans-inactivation of the

paternally expressed protein coding genes (*DLK1* and *PEG11*) by maternally expressed ncRNAs (*GTL2, anti-PEG11, RIAN, MIRG*, and embedded miRNAs and snoRNAs) in phenotypically normal *CLPG/CLPG* animals. We previously showed that *PEG11* is inactivated (sliced) by miRNAs processed from the maternally expressed *anti-PEG11* [11]. We herein aim to address the possibility that DLK1 protein expression is also modulated by miRNAs derived from the imprinted domain by measuring affinities of these miRNAs for *DLK1* in cultured cells.

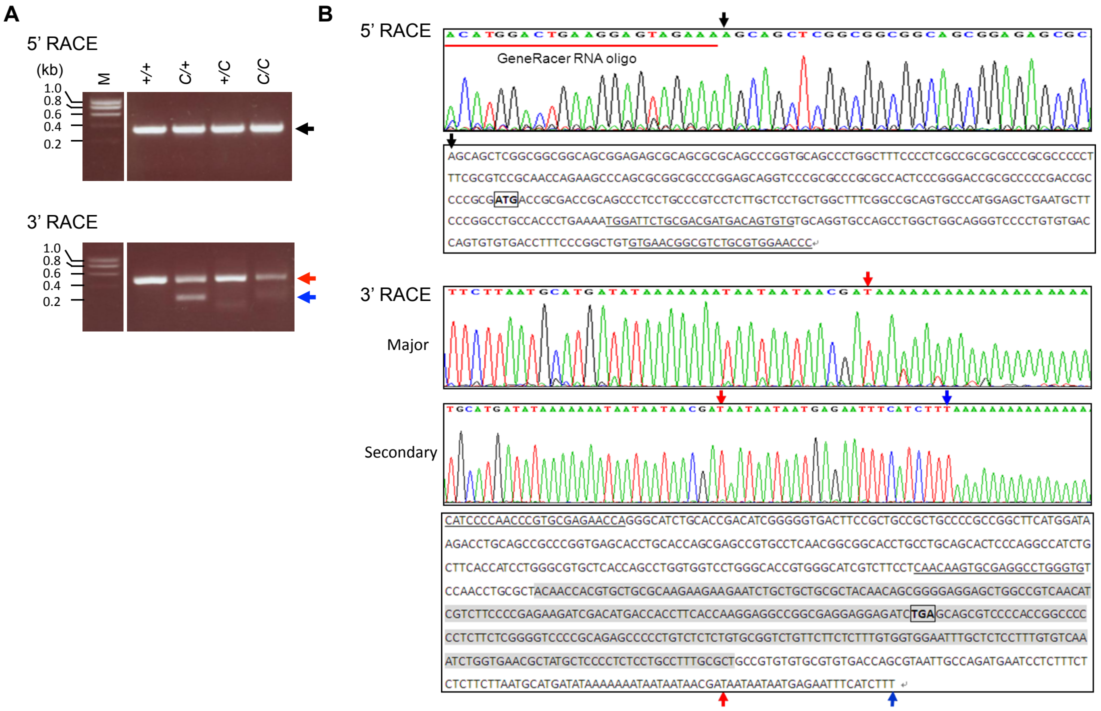

Supplemental Figure 2: Characterization of the 5' and 3' ends of ovine *DLK1* transcripts in ovine skeletal muscle. (**A**) Agarose gel electrophoreses of 5' RACE and 3' RACE PCR products obtained from *longissimus dorsi* skeletal muscle RNA of sheep with the four *CLPG* genotypes (*+/+*, $CLPG^{Mat}$*/+*, *+/*$CLPG^{Pat}$, *CLPG/CLPG*) using the GeneRacer Kit (Invitrogen). RT-PCRs were carried out using the GeneRacer 5' and ovine *DLK1*-specific 5' GSP-A primers (5' RACE), and the GeneRacer 3' and ovine *DLK1*-specific 3' GSP-S primers (3' RACE), followed by nested PCRs using the GeneRacer 5' nested and ovine *DLK1*-specific 5' NGSP-A primers (5' RACE), and the GeneRacer 3' nested and ovine *DLK1*-specific 3' NGSP-S primers (3' RACE). The major 5' and 3' RACE PCR products (black and red arrows, respectively) were directly sequenced. The secondary 3' RACE PCR product (blue arrow) was cloned into the TA cloning vector pCR2.1 (Invirogen) and then sequenced. M, DNA molecular weight marker. Primer sequences are available in Suppl. Table 1. (**B**) Representative chromatograms obtained by sequencing the major 5' RACE, and major and secondary 3' RACE PCR products, and corresponding summary sequences. The linker sequence (GeneRacer RNA Oligo) used for 5' RACE is underlined in red. The deduced transcription start site and the major and secondary polyadenylation sites are shown by black, red, and

blue arrows, respectively. The shaded (grey) sequence (in the lowest panel) corresponds to the segment that was not observed in the secondary 3' RACE PCR product. This splicing variant is most likely a PCR artifact, as the variant (i) was not observed in the primary 3' RACE PCR products, (ii) is not characterized by canonical splicing donor and acceptor sites, and (iii) was not observed in RNA sequencing data from sheep of the four genotypes (data not shown). The start and stop codons are boxed, and the *DLK1*-specific primers (5' GSP-A, 5' NGSP-A, 3' GSP-S, 3' NGSP-S) underlined in black.

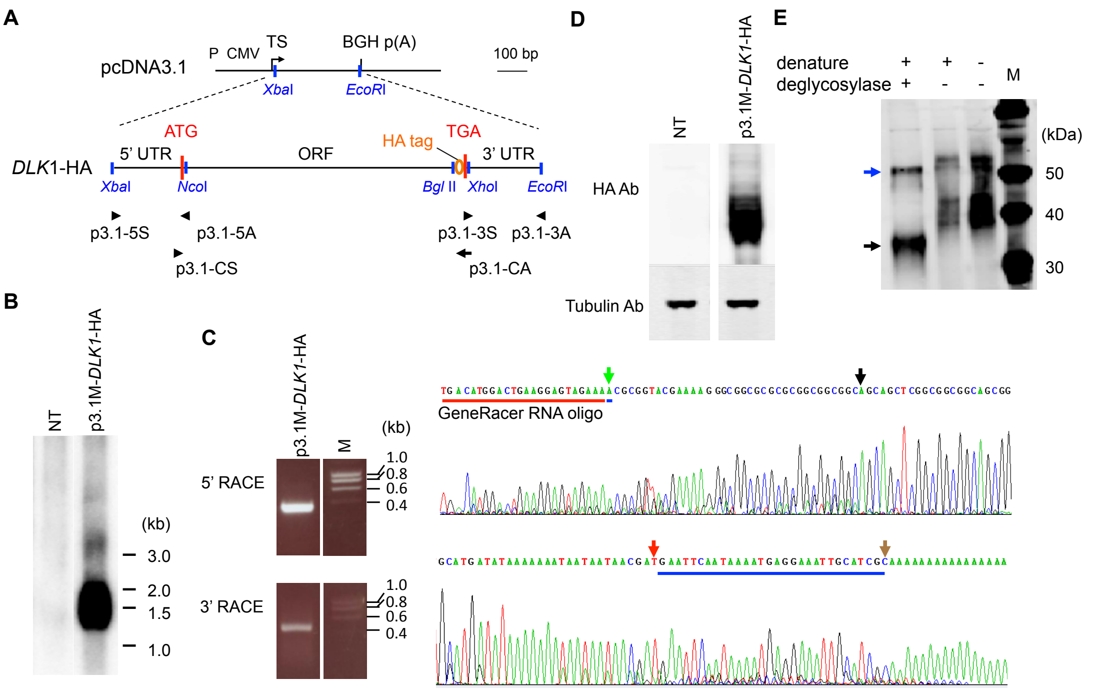

Supplemental Figure 3: Construction and characterization of the p3.1M-*DLK1*-HA vector expressing ovine *DLK1*. **(A)** Main features of the p3.1M-*DLK1*-HA expression vector. The pcDNA3.1(-) vector was modified such as to introduce an *Xba*I site adjacent to the vector-specific transcription start site (TS) and an *EcoR*I site upstream of the bovine growth hormone polyadenylation signal (BGH p(A)), resulting in the removal of the intervening 283 bp sequence from the vector (p3.1M). In total 1,405 bp of DNA fragment (*DLK1*-HA) corresponding to 209 bases of the ovine *DLK1* 5' UTR, 927 bases of the *DLK1* ORF (C2 isoform), 27 bases of carboxy-terminal HA tag, 6 bases of *Xho*I recognition sequence, and 236 bases of the *DLK1* 3' UTR was assembled from three PCR products amplified with three primer pairs; p3.1-5S and -5A, p3.1-CS and -CA, and p3.1-3S and -3A. By utilizing *Xba*I, *Nco*I, *Xho*I, and *EcoR*I recognition sites, these fragments were sequentially ligated into the *Xba*I-*EcoR*I sites of the p3.1M vector. Expression of the *DLK1*-HA was driven by the Cytomegalovirus enhancer-promoter (P CMV). ATG, start codon; TGA, stop codon. Primer information and RNA sequence transcribed from the vector are described in Suppl. Table 3 and Suppl. Text 1, respectively. **(B)** Northern blot analysis of RNA from COS1 cells transfected with the p3.1M-*DLK1*-HA using a probe (857 bp) obtained by double

digestion (*Nco*I and *Bgl*II) of the plasmid p3.1M-*DLK1*-HA. Size of the transcript expected to be expressed from the vector is ~1.4 kb. NT corresponds to non-transfected cell control. **(C)** Identification of transcription start and polyadenylation sites of the p3.1M-*DLK1*-HA-derived transcript. Agarose gel electrophoreses (left panels) of 5' RACE and 3' RACE PCR products using RNA from COS1 cells transfected with the p3.1M-*DLK1*-HA vector and chromatograms (right panels) obtained by direct sequencing of the PCR products. The black and green arrows mark major transcription start sites identified in sheep muscle RNA (Suppl. Fig. 2) and p3.1M-*DLK1*-HA-derived transcripts, respectively. The red and brown arrows mark predominant polyadenylation sites identified in sheep muscle RNA (Suppl. Fig. 2) and p3.1M-*DLK1*-HA-derived transcripts, respectively. The sequences underlined in red and blue correspond to the GeneRacer RNA Oligo (used as a 5' linker) and vector derived sequences, respectively. **(D)** Western blot analysis of protein extracts from COS1 cells transfected with the p3.1M-*DLK1*-HA vector using anti-HA antibodies (HA Ab) for detecting DLK1 and anti-Tubulin antibodies (Tubulin Ab) as loading control. NT corresponds to non-transfected cell control. **(E)** Effect of deglycosylation of DLK1 protein on its molecular weight. We used the Protein Deglycosylation Mix (New England Biolabs) to remove all *N*-linked and simple *O*-linked glycans, as well as some complex *O*-linked glycans from proteins. Total proteins used in (D) were heat-denatured, treated with a mixture of deglycosylation enzymes, and subjected to Western blot analysis using anti-HA antibodies. Aliquot of the cell extracts without any treatment and treated similarly without glycosylases were loaded for comparisons. The black arrow indicates presumed deglycosylated DLK1 protein with a predicted molecular weight ~34.13 kDa. Nature of the larger band pointed with the blue arrow remains unknown. Post-translational modifications of DLK1 by *N*- and *O*-linked glycosylation, phosphorylation, and ubiquitination have been reported [30]. (B-E) M, molecular weight marker.

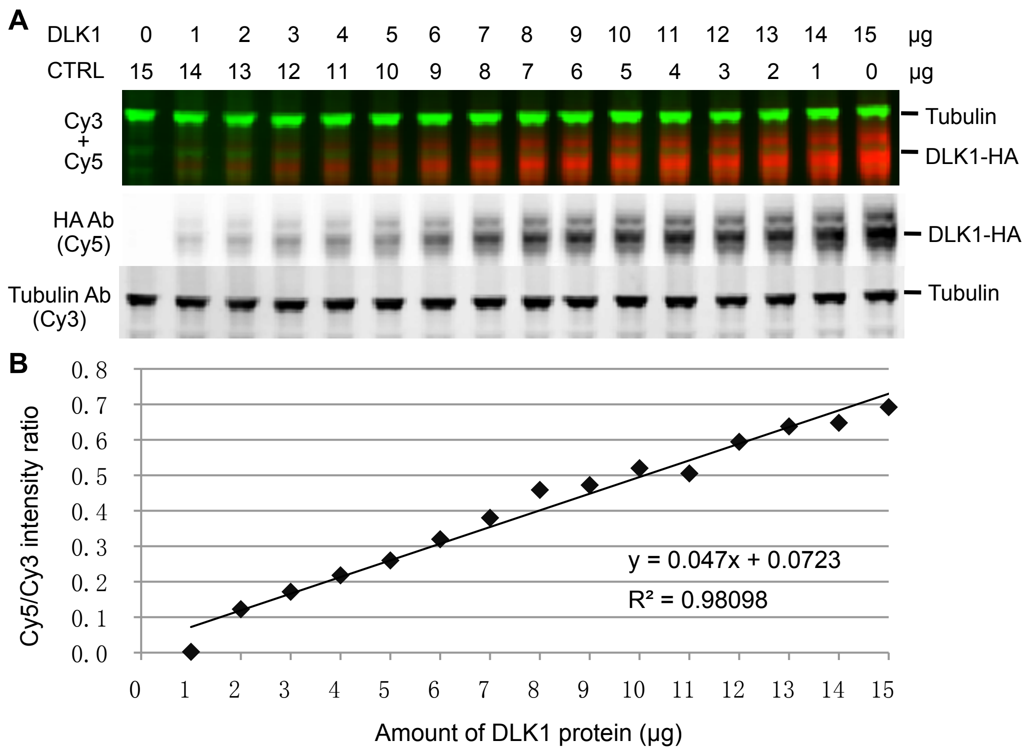

Supplemental Figure 4: Quantitativeness of Western blot analysis of ovine DLK1 protein. **(A)** Proteins were extracted from COS1 cells transfected with either the p3.1M-*DLK1*-HA (DLK1) or pcDNA3.1(-) vector (CTRL), along with a negative control mimic miRNA (Ambion AM17110). The DLK1-containing protein (DLK1) was mixed with an incremental amount of the control protein (CTRL) as shown on the top. In total 15 µg of protein per well was subjected to dual fluorescent Western blot analysis. We first incubated with a mixture of primary antibodies (rabbit polyclonal anti-HA tag and mouse monoclonal anti-α-Tubulin antibodies) followed by incubating with a mixture of secondary antibodies (Cy5-conjugated anti-rabbit IgG and Cy3-conjugated anti-mouse IgG antibodies) to detect DLK1-HA and Tubulin proteins simultaneously. The Cy5 and Cy3 fluorescent intensities were measured with a Typhoon phosphoimager and the ImageQuant TL software (GE Healthcare). Two-color fluorescence and gray-scale single channel images are shown. **(B)** Ratios of Cy5 over Cy3 intensities (DLK1/Tubulin) were plotted against the corresponding amounts of protein. Linear regression analysis was performed and its linear predictor function and coefficient of determination (R^2) are shown.

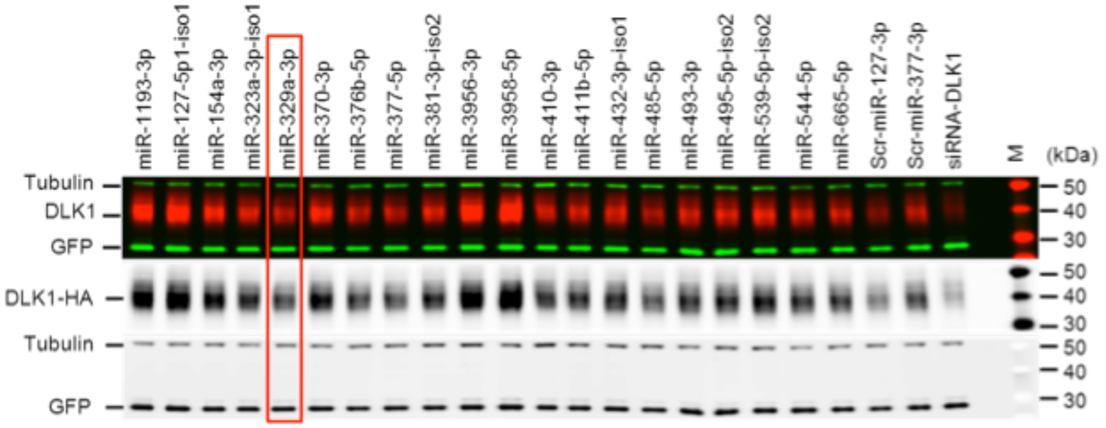

Supplemental Figure 5: Dual fluorescent Western blot to measure DLK1 amounts. Representative image of dual fluorescent Western blot analysis. COS1 cells were co-transfected with the p3.1M-*DLK1*-HA and pcDNA3-*GFP* vectors along with a synthetic mimic miRNA indicated above the image. In a gel, we loaded twenty samples transfected with mimic miRNAs in the *DLK1-GTL2* domain and three controls (Scr-miR-127-3p, Scr-miR-377-3p, siRNA) with a molecular marker. Proteins were transferred to a PVDF membrane and sequentially incubated with antibodies recognizing HA tag, GFP, and endogenous Tubulin. As anti-HA tag antibodies were rabbit polyclonal, they were labeled with Cy-5 conjugated secondary antibodies. On the other hand, anti-GFP and anti-α-Tubulin antibodies were mouse monoclonal and then labeled with Cy-3 conjugated secondary antibodies (see Methods for details). The membrane was scanned with a Typhoon 9400 scanner (GE healthcare). Band intensities corresponding to HA-tagged DLK1, GFP, and Tubulin were quantified using the ImageQuant TL software (GE healthcare). For normalization, the band intensities were first divided by the corresponding membrane-specific averages, yielding "relative" DLK1, GFP, and Tubulin amounts. The relative DLK1 amounts were then divided by either the relative GFP or Tubulin amounts, yielding "corrected" DLK1 amounts. We performed more than four independent transfections of cells for each mimic miRNA, and ran more than two independent Western blot experiments per transfection. Result for miR-329a-3p that showed the strongest inhibiting effect on DLK1 expression is boxed in red.

DLK1 ORF, 6-mer match position: 332-337
Miranda Score: 120, Energy: -11.5 kCal/Mol

```
   oar-329a-3p:          3' tttttccaattggtcCACACAa 5'
                                           ||||||
   ovine DLK1:           5' tctgcgacgatgacaGTGTGTg 3'

   sheep        CTGAAAATGGATTCTGCGACGATGACAGTGTGTGCAGGTG
   human        C--AAAATGGATTCTGCGAGGATGACAATGTTTGCAGGTA
   mouse        C--AGTATGGATTCTGCGAGGCTGACAATGTCTGCAGGTA
   dog          C--AAAATGGATTCTGCGAGGATGACAATGTTTGCAGGTA
   elephant     AT--ACATGGATCCTGCGTGGATGAGAACATCTGCAGGTG
                      ****** *****  * *** *   * *******
```

DLK1 ORF, 6-mer match position: 378-383
Miranda Score: 121, Energy: -18.1 kCal/Mol

```
   oar-329a-3p:         3' tttttcCA-ATTGGTCCACACAa 5'
                                 || |:|||  ||||||
   ovine DLK1:          5' tcccctGTGTGACC-AGTGTGTg 3'

   sheep        TGGCTGGCAGGGTCCCCTGTGTGACCAGTGTGTGACCTTTC
   human        TGGCTGGCAGGGTCCCCTTTGTGACCAGTGCGTGACCTCTC
   mouse        TGGCTGGGAGGGTCCCCTCTGTGACAAGTGTGTAACTGCCC
   dog          TGGCTGGCAGGGTCCCCTGTGTGACCAGTGCGTGACCTTTC
   elephant     TGGCTGGCAGGGTCCCCTGTGTGACCATTGCGTGCCCTCTC
                ******* ********** ****** * ** **  *    *
```

DLK1 3′ UTR, 6-mer match position: 1322-1327
Miranda Score: 120, Energy: -12.8 kCal/Mol

```
   oar-329a-3p:           3' tttttccaattggtcCACACAa 5'
                                            ||||||
   ovine DLK1:            5' tgcctttgcgctgccGTGTGTg 3'

   sheep         GCTCCCCTCTCCTGCCTTTGCGCTGCCGTGTGTGCGTGTGAC
   human         GCTTACATATATTGTCTTTGTGCTGCTGTGTG--------AC
   mouse         GCTTACATATATTGTCTT-GTGTTGCTGTGTG--------CC
   dog           GCTTCCATATATTATCTTTGTGTTGCTCTGTG--------AC
   cow           GCTCCCCTCTCCTGCCTTTGCGCTGCCGTGTGTG--TGCAAC
   elephant      GCTTGCATTTATTGTCTTTGTGTCGCTGTGTG--------AT
                 ***  * * *  *  *** * *  **  ****
```

Supplemental Figure 6: Conservation around oar-miR-329a-3p 6-mer seed matches on *DLK1*. MiR-329a-3p that showed the most inhibiting effect on DLK1 amount has three 6-mer seed matches [15] in ovine *DLK1* (two in CDS and one in 3' UTR). Positions of the seed match in the transcript sequence from the p3.1M-*DLK1*-HA (Suppl. Text 1), miRNA-target duplex provided by Miranda software [16] and its score and minimum free energy, and multiple alignments surrounding the predicted target site of indicated mammals obtained from the UCSC genome browser Multiz Alignment Cons 46-Way [32] are shown.

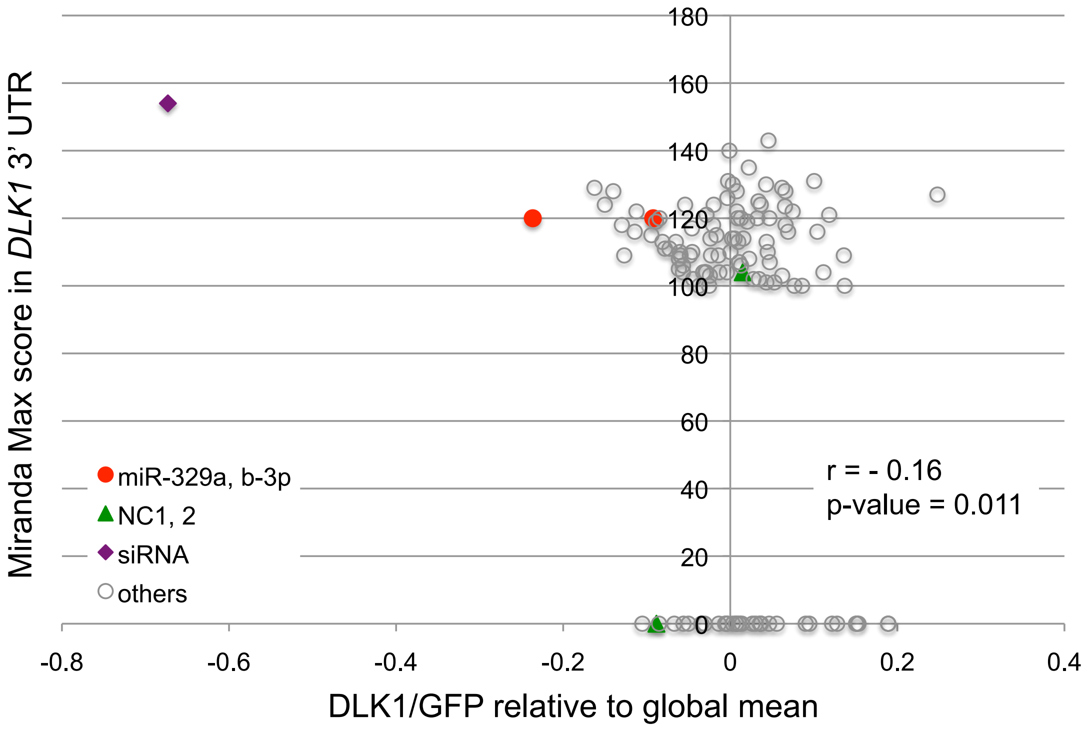

Supplemental Figure 7: Weak correlation between miRNA-target affinity score and DLK1. Miranda scores (Max-score in ovine *DLK1* 3' UTR) were plotted against the corrected DLK1 values (DLK1/GFP) relative to the global mean. Data for miR-329a-3p and closely related miR-329b-3p, and three controls are highlighted as described in left inset. Correlation coefficient (calculated without controls) and its p-value are shown (right inset). From the five score (Miranda Tot-Score, Max-Score, Tot-Energy and Max-Energy scores [16] and Grimson seed match score [15]) x *DLK1* segments (5' UTR, ORF, 3' UTR, full length) combinations tested, this is the one that gave the most significant, yet not very convincing correlation.

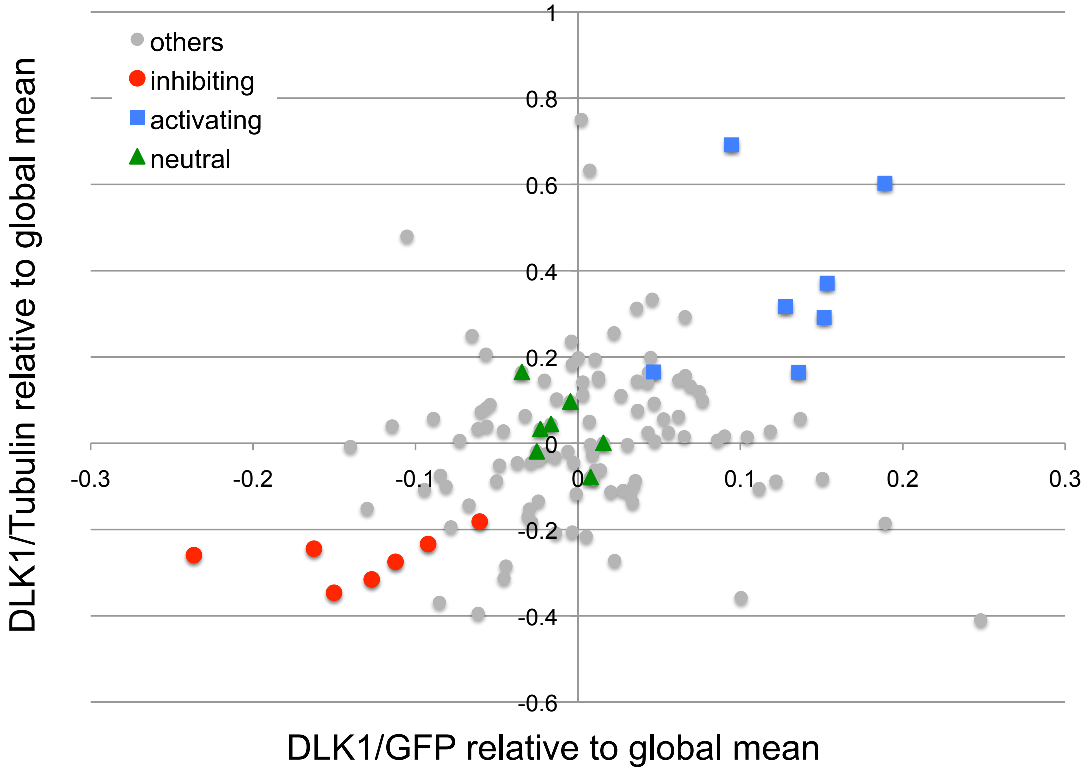

Supplemental Figure 8: MiRNAs used in multiple-miRNA transfection test. We selected seven miRNAs that stably exhibited inhibiting, activating, or neutral effects on DLK1 protein expression (shown in red, blue and green, respectively). List of the miRNAs is in Suppl. Table 2. Result of the multiple-miRNA transfection test using a mixture of the seven miRNAs is shown in Fig. 1C.

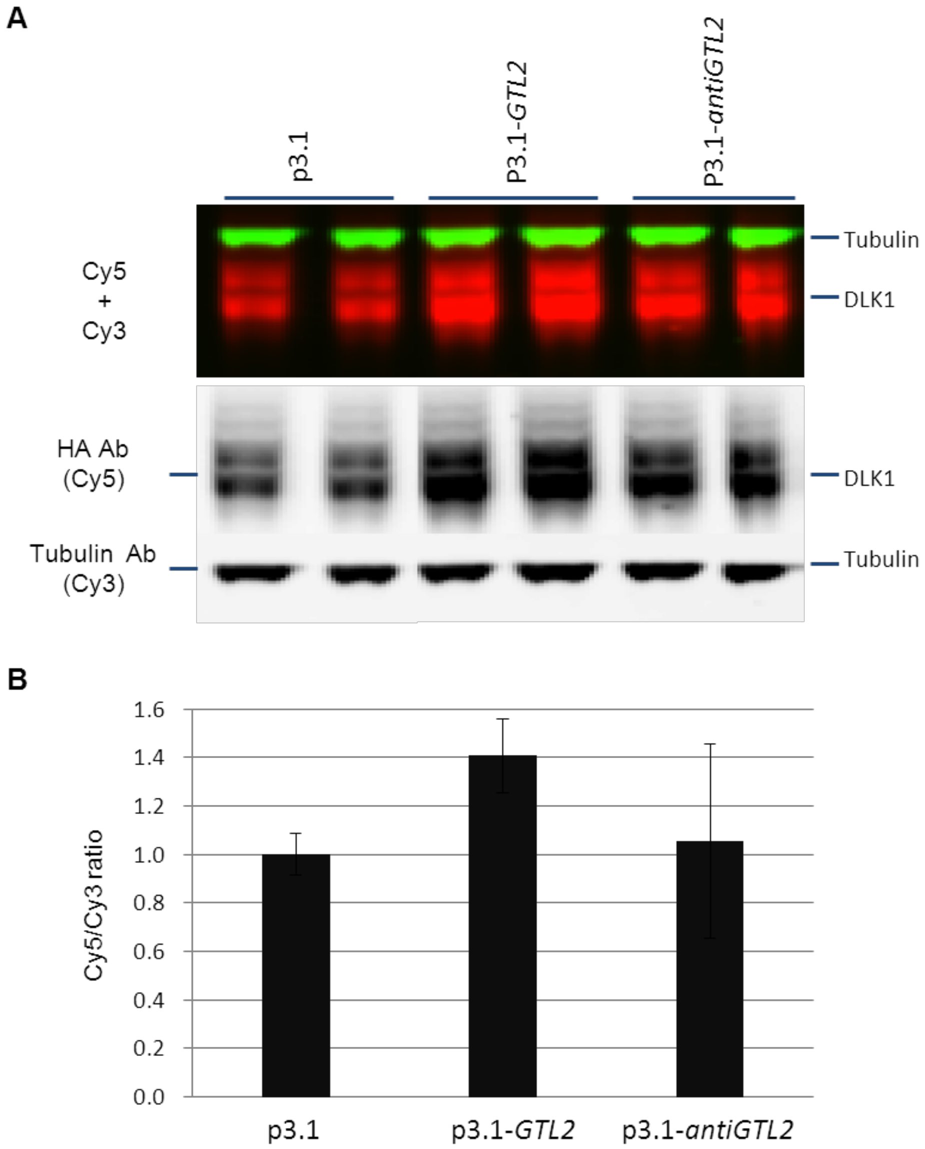

Supplemental Figure 9: Effect of *GTL2* lncRNA expression on DLK1 amount. (A) Dual fluorescent Western blot using proteins from COS1 cells transfected with the p3.1M-*DLK1*-HA along with either an empty pcDNA3.1 (p3.1), *GTL2*-expressing vector (p3.1-*GTL2*), or a vector with the same *GTL2* sequence but in an antisense direction (p3.1-*antiGTL2*). DLK1-HA and Tubulin protein levels were estimated by the dual fluorescent Western blot analysis as described before. (B) Means of corrected DLK1 amounts (Cy5 over Cy3 intensities) with standard deviations for two biological replicates are shown.

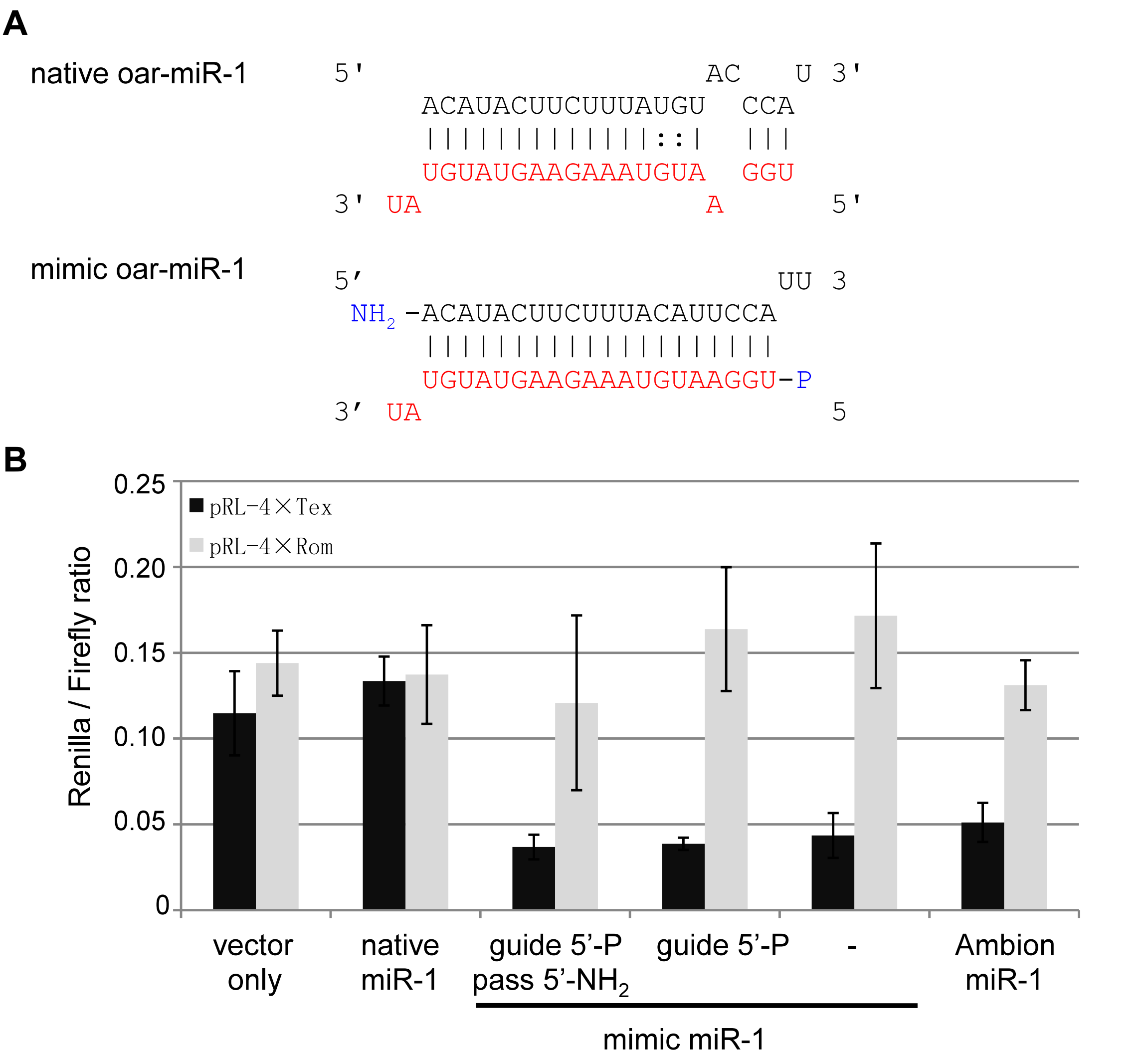

Supplemental Figure 10: Functionality of synthetic mimic miRNAs. (A) Structure of tested double stranded small RNAs (dsRNA). Native oar-miR-1 was synthesized according to the endogenous ovine sequences (mature and star miR-1), hence containing two wobble base pairs, a mismatch, and a bulge in its dsRNA. Mimic oar-miR-1 has a siRNA-like structure in which its passenger strand is completely complementary to the oar-miR-1 mature sequence with 2-nt overhangs at both 3' ends. Oar-miR-1 mature (= guide strand) sequence is shown in red. Tested 5' end modifications (5' phosphate on guide strand and 5' amine on passenger strand) are shown in blue. (B) Dual luciferase assay (Promega) was performed using two renilla luciferase (RL) reporter vectors essentially as described in Takeda et al. [17]. The pRL-4xTex vector contains four tandem copies of miR-1 target sites in its 3' UTR, while corresponding four repetitions of

the pRL-4xRom do not. We co-transfected COS1 cells with one of the pRL reporter vectors, a firefly luciferase-expressing pGL3 vector for normalization of transfection efficiency, and a series of synthetic dsRNAs. Means of normalized RL intensities (ratio of RL over firefly luciferase intensities) with standard deviation for three independent experiments are shown. Co-transfection was done either without addition of dsRNA (vector only), or the miR-1 dsRNA with native structure (native), or the perfectly complementary mimic miR-1 dsRNA with 5' phosphate modification on guide strand (guide 5'-P) and 5' NH_2 modification on passenger strand (pass 5'-NH_2), or the same mimic miR-1 dsRNA only with the guide 5'-P, or the same mimic miR-1 dsRNA without any 5' modification (-), or proprietary miR-1 precursor purchased from Ambion for comparison.

Supplemental Text 1: RNA sequence transcribed from the p3.1M-*DLK1*-HA expression vector.

1) 5' UTR (210 bases in total) (one base at the 5' end coloured in gray is originated from the expression vector)(The transcription start site observed in ovine skeletal muscle is boxed).

ACGCGGUACGAAAAGGGCGGCGCGCGGCGGCGGCAGCAGCUCGGCGGCGGCAGCG
GAGAGCGCAGCGCGCAGCCCGGUGCAGCCCUGGCUUUCCCCUCGCCGCGCCCGCGC
CCCCUUUCGCGUCCGCAACCAGAAGCCCAGCGCGGCGCCCGGAGCAGGUCCCGCGCCC
GCGCCACUCCCGGGACCGCGCCCCCGACCGCCCCGCG

2) ORF (954 bases in total; 927 bases of the ovine *DLK1* C2 isoform and 27 bases of HA-tag at the carboxyterminus)(a start codon ATG and a stop codon TGA are shown in bold, HA-tag sequence is boxed, endogenous restriction enzyme recognition sites of *Nco*I CCATGG and *Bgl*II AGATCT are underlined).

AUGACCGCGACCGCAGCCCUCCUGCCCGUCCUCUUGCUCCUGCUGGCUUUCGGCCGC
AGUGC<u>CCAUGG</u>AGCUGAAUGCUUCCCGGCCUGCCACCCUGAAAAUGGAUUCUGCGAC
GAUGACAGUGUGUGCAGGUGCCAGCCUGGCUGGCAGGGUCCCCUGUGUGACCAGUG
UGUGACCUUUCCCGGCUGUGUGAACGGCGUCUGCGUGGAACCCUGGCAGUGCAUCU
GCAAGGACGGCUGGGACGGACACCUCUGUGACCUAGACAUCCGGGCUUGCACCUCGA
CCCCCUGUGCCAACAACGGCACCUGCCUGGACCUCGAUGACGGCCAGUACGAGUGCU
CCUGUGUCCCCGGGUUCUCGGGAAAGGAUUGCCAGGAAAUGGACGGGCCCUGCGUG
GUGAAUGGCUCGCCCUGCCAGCACGGAGGCAGCUGCGUGGACGAUGAGGGCCGGGCC
CCCCACGCCGUCUGCCUGUGCCCCCCUGGCUUCUCGGGCAACUUCUGCGAGAUCAUG
ACCAACAGCUGCAUCCCCAACCCGUGCGAGAACCAGGGCAUCUGCACCGACAUCGGG
GGUGACUUCCGCUGCCGCUGCCCCGCCGGCUUCAUGGAUAAGACCUGCAGCCGCCCG
GUGAGCACCUGCACCAGCGAGCCGUGCCUCAACGGCGGCACCUGCCUGCAGCACUCCC
AGGCCAUCUGCUUCACCAUCCUGGGCGUGCUCACCAGCCUGGUGGUCCUGGGCACCG
UGGGCAUCGUCUUCCUCAACAAGUGCGAGGCCUGGGUGUCCAACCUGCGCUACAACC
ACGUGCUGCGCAAGAAGAAGAAUCUGCUGCUGCGCUACAACAGCGGGGAGGAGCUG
GCCGUCAACAUCGUCUUCCCCGAGAAGAUCGACAUGACCACCUUCACCAAGGAGGCC
GGCGAGGAGG<u>AGAUCU</u>ACCCAUACGACGUCCCAGACUACGCU**UGA**

3) 3' UTR (272 bases in total; 6 bases of an exogenously introduced *Xho*I recognition sequence, 236 bases of the ovine *DLK1* 3' UTR and 30 bases derived from the vector)(the *Xho*I site is underlined, the major polyadenylation site

observed in ovine skeletal muscle is boxed, the sequence originated from the vector is coloured in gray, a native non-canonical polyadenylation signal on the ovine *DLK1* gene and the bovine growth hormone polyadenylation signal on the vector are shown in bold).

CUCGAGGCAGCGUCCCCACCGGCCCCCCUCUUCUCGGGGUCCCCGCAGAGCCCCCUGU
CUCUCUGUGCGGUCUGUUCUUCUCUUUGUGGUGGAAUUUGCUCUCCUUUGUGUCAA
AUCUGGUGAACGCUAUGCUCCCCUCUCCUGCCUUUGCGCUGCCGUGUGUGCGUGUG
ACCAGCGUAAUUGCCAGAUGAAUCCUCUUUCUCUCUUCUUAAUGCAUGA**UAUAAA**
AAAAUAAUAAUAACGAUGAAUUC**AAUAAA**AUGAGGAAAUUGCAUCGC

Supplemental Text 2: Ovine *GTL2* sequence in the p3.1-*GTL2* expression vector.

GGAGAGCTGGGATGGAGCGCGCCTTGGCCCGCTGGCCTGGGCCGCGGCTCCTCCGGAG
AGCCCGGGCGCCCACGCGAGAACCTCCCTACCCGGGTCTGTCTTCAAGGATGACGCCT
TCCGTCTGCCTTCCTGCCACCAAGGACCACCTGTGGATGACGAGCTGATGCCCAGAGG
TCGCCAGGAGCAGGGGTTCCTGGATCCCACCAGCAAACAAGGCAACCACCGGGCAGTG
ACCGCCCCCCCCAGGCCGCCGAAGGATGAAGAGGACTCGAACTGACCAGCCCGCTGTC
CCTCTTGGCTAAGTGTTGAAACCAGCGCCCTAGTGAGGGGGCGCTGGTTAACCTTGG
ACTTTCGATGTGCTTCTGCCTCAGTGCTTTGCTTTTCTATGTGTGTCTGCTTCTGTTA
ACCTTCCATCAACCTGGAGGTTTCACCATCCCCAGGGAGGCGTCAGTCTTTCTCAGTC
CACCCCGCCATTACTCCCAGGATGGTCTGAAAGGAAGGGTCCCTTTGGGAACTTCTCA
GGAGGGAGACCTGGGCCAAGGGCTCGACCAGCATCTCCCTGGCAATTCCAAGGCCCAG
GGTGGGCTTCTGGAAGGAGCATGATTCCAGAGGCCATGCTACTGAACTGCCAGGGGC
ATCCCCACCTCCTCCAGATCTCCTCATCCTTTCTGGGGAACGGGATCGAGAAAAGCAG
CCTCCCAGGGTGTTGTGAGTGTTGGTCCGATTCTGGGAAGCCGGTAAGGCCGGGCAT
AGACGAGGCGGTCAGTAAATGTTTTTTCCCAAACAGGTTGGTCGACCACGTCCCCCTC
GCCCCTCTCACCTGTCTCACGCTTCTCGTTTATTCTCCCACAGCGCTCCGGACAACCCC
AGTCCGCAGGGCTCTCGGTGTACCAGCCCCGCCTCCTGTACAGCCTATCTACATCCGC
TGTCTTCCTTCCTCACCTTCCGTTTCCCCTCCAACCCACCGCTTCGTGGTCTCCTCAGG
TCCATTGAACTCCACCAGCTCTAAGGAGCCCCGGCGGCCCCGGATCGTGAACGCCTGG
ACGGAGGAGGTGGAGGAGCTACCCTGGAGCAGTACGTCTTCCGGAGGGTCACCCGGC
GTCTCGCGCCCGCCCCGAGCCGCGATGCTGCTGCCTGGATTGGGCCGAAGCCATCATC
TGGAATCCTCCGTGGTCCAGGACCTCGAACCCGCCACCCCAGCACCCTCTAGCTCCAT
AATCCCTCCACAGCCACAGGGGACACCTGCACGCGTTCCCACGGGACAGGCTGGACCC
AAAGACTCTGGACCTGGGCTGACCCCCCTGTGCGGAGGGGCGTGTCCTCCCCGCCTCC
CCTCCCTCTGACTGATGGACTTCACTGATCTGAGTGTCAGACACGCGGGCCGGACCCC
CTCCCTGCCCCTCACGAACCCTGAGACTTGGGAGAGCGCCGGGCCTCGCTGTCCACAT
CTGAGAAATGGGCTCGGACACCTACCTCACAGGGCTGTTGTGAGGAGCCTGTGCCAG
GGTCGGAAAGCACCCTCCCCCAGTGGCGGGGGCGGGGCTTGGCACGGCCCATTGTGCT
CTCAATAAATATGTTTCTTGTCTTAACAAAAACA
